# IGHV基因使用片段在伴有骨髓浸润的晚期滤泡性淋巴瘤患者中的预后价值

**DOI:** 10.3760/cma.j.cn121090-20251207-00575

**Published:** 2026-06

**Authors:** 永乐 李, 睿泽 陈, 楠 陈, 彤璐 邱, 泓妤 沈, 晖 金, 纯 乔, 华渊 朱, 磊 范, 卫 徐, 建勇 李, 文瑜 施, 祎 缪, 奕 夏

**Affiliations:** 1 南京医科大学第一附属医院，江苏省人民医院血液科，南京 210029 Department of Hematology, The First Affiliated Hospital of Nanjing Medical University, Jiangsu Province Hospital, Nanjing 210029, China; 2 南通大学附属医院血液科，南通 226001 Department of Hematology, Affiliated Hospital of Nantong University, Nantong, 226001, China

**Keywords:** 淋巴瘤，滤泡性, 骨髓浸润, IGHV3-48, POD12, Lymphoma, follicular, Bone marrow infiltration, IGHV3-48, POD12

## Abstract

**目的:**

探讨免疫球蛋白重链可变区（IGHV）基因使用片段特征在伴有骨髓浸润的高危滤泡性淋巴瘤（FL）患者中的预后价值。

**方法:**

回顾性分析2017年8月至2024年11月于江苏省人民医院确诊为伴有骨髓浸润的33例FL患者的临床资料。

**结果:**

33例FL患者中，32例（97.0％）患者存在IGHV突变，中位胚系基因符合率为90.7％。IGHV3家族呈现出显著的优势分布（23例，69.7％），其中以IGHV3-48（9例，27.3％）、IGHV3-23（4例，12.1％）和IGHV3-66（4例，12.1％）为主要使用的基因片段。与既往文献报道相比，本队列中IGHV3-48基因的使用率（27.3％）高于慢性淋巴细胞白血病（3.8％）、脾边缘区淋巴瘤（0）、华氏巨球蛋白血症（2.2％）等其他B细胞淋巴瘤（*P*值均<0.05）。单因素分析结果显示，使用IGHV3-48基因的FL患者高乳酸脱氢酶水平［66.7％对13.6％；*OR*＝12.7（95％ *CI*：1.6～102.3），*P*＝0.017］及FL国际预后指数2评分≥3分［88.9％对33.3％；*OR*＝15.0（95％ *CI*：1.6～142.2），*P*＝0.018］比例高。此外，使用IGHV3-48基因片段的患者也表现出在12个月内疾病进展（POD12）风险升高的趋势［44.4％对12.5％；*OR*＝4.8（95％*CI*：0.8～28.9），*P*＝0.087］。

**结论:**

IGHV3-48基因的使用对于早期预测具有骨髓浸润特征的FL患者预后可能具有一定的参考价值。

## Abstract (en)

**Objective:**

To investigate the prognostic value of immunoglobulin heavy chain variable region（IGHV）gene segment usage characteristics in high-risk follicular lymphoma（FL）patients with bone marrow infiltration.

**Methods:**

The clinical data of 33 patients of FL with bone marrow infiltration diagnosed at Jiangsu Province Hospital from August 2017 to November 2024 were collected and retrospectively analyzed.

**Results:**

Among the 33 enrolled FL patients, 32（97.0％）had mutated IGHV, with a median germline identity of 90.7％. The IGHV3 family was predominant（23 cases, 69.7％）, with IGHV3-48（9 cases, 27.3％）, IGHV3-23（4 cases, 12.1％）, and IGHV3-66（4 cases, 12.1％）as the most frequent segments. Consistent with prior reports, the frequency of IGHV3-48 usage in our cohort（27.3％）was higher than that observed in other B-cell lymphomas, including chronic lymphocytic leukemia（3.8％）, splenic marginal zone lymphoma（0）, and Waldenström macroglobulinemia（2.2％）（all *P* value<0.05）. Univariate analysis showed that FL patients using the IGHV3-48 gene exhibited higher lactate dehydrogenase levels（66.7％ *vs* 13.6％; *OR*＝12.7, 95％ *CI*: 1.6–102.3, *P*＝0.017）and a higher proportion of FLIPI-2 score ≥3（88.9％ *vs* 33.3％; *OR*＝15.0, 95％ *CI*: 1.6–142.2, *P*＝0.018）. Furthermore, patients using the IGHV3-48 gene segment also demonstrated a trend toward an increased risk of disease progression within 12 months（POD12）（44.4％ *vs* 12.5％; *OR*＝4.8, 95％ *CI*: 0.8–28.9, *P*＝0.087）.

**Conclusion:**

The usage of the IGHV3-48 gene may have certain reference value for early prognostic prediction in FL patients with bone marrow infiltration.

滤泡性淋巴瘤（FL）是最常见的惰性非霍奇金B细胞淋巴瘤，占非霍奇金淋巴瘤的20％～25％[1]–[2]。随着治疗方案的不断优化，FL的预后获得显著改善，但仍有约20％的患者在初始治疗后出现早期复发，且每年约2％的FL会转化为侵袭性淋巴瘤，是患者生存期缩短的主要原因[3]–[4]。然而，目前尚缺乏能够早期预测疾病复发和转化的可靠生物标志物[5]。

免疫球蛋白基因重排是B细胞淋巴瘤的克隆性标志。免疫球蛋白基因检测采用Sanger测序，成本低廉，且免疫球蛋白基因使用特征在疾病进程中保持稳定不变，是早期判断预后的可靠标志物。免疫球蛋白重链可变区（IGHV）基因的突变状态与预后密切相关，这一现象在慢性淋巴细胞白血病（CLL）、脾边缘区淋巴瘤（SMZL）和套细胞淋巴瘤（MCL）中均得到证实[6]–[8]。然而关于FL中IGHV基因使用特征的研究较为有限。已有研究发现IGHV基因的使用与FL的预后及组织学转化相关[9]–[10]。骨髓浸润作为FL的不良预后因素，已被纳入FL国际预后指数（FLIPI）2、PRIMA-PI等关键预后模型[11]–[12]。目前国内尚未见针对FL患者IGHV基因使用特征的报道，而对其中伴有骨髓浸润的高危亚组则更缺乏关注。因此，本研究旨在回顾性分析该群体中IGHV基因使用特征，以期为早期识别高危FL提供线索。

## 病例与方法

1. 病例：本研究为回顾性队列研究，纳入2017年8月至2024年11月期间在江苏省人民医院确诊的FL患者。纳入标准：①经组织病理学检查（形态学及免疫组化）确诊为FL，诊断依据世界卫生组织分类及《中国滤泡性淋巴瘤诊断与治疗指南（2023年版）》[13]，分期标准按照2014年版Lugano分期系统[14]；②骨髓活检证实存在骨髓浸润（Ann Arbor分期为Ⅳ期）；③临床资料完整，可获取年龄、性别、美国东部肿瘤协作组（ECOG）评分、FLIPI评分及治疗方案等信息。共纳入33例FL患者，回顾患者的既往病历，收集临床信息。本研究由江苏省人民医院伦理委员会批准（批件号：2024-SR-1021）。

2. 免疫球蛋白重排检测与鉴定：诊断为FL时，经患者知情同意后收集适量骨髓样本，采用免疫球蛋白重链体细胞超突变测定v2.0试剂盒（美国InVivoScribe公司）进行聚合酶链式反应（PCR）扩增。PCR扩增首先使用leader引物，仅在leader引物[15]–[16]未能获得可靠扩增时，采用重链可变区框架区1（VH FR1）引物。使用IMGT/VQUEST工具（版本3.3.0）对比测序结果并记录IGHV、免疫球蛋白重链多样性（IGHD）、免疫球蛋白重链连接区（IGHJ）基因使用和体细胞超突变状态（SHM）。本研究33例患者中，27例（81.8％）使用leader引物扩增，6例（18.2％）使用VH FR1区引物扩增。根据与最接近的IGHV胚系基因的同源性百分比，将≥98％同源性定义为无突变，<98％定义为突变[17]。

3. 随访：随访截止时间为2025年1月25日，采取病历查阅及电话方式进行随访。12个月内疾病进展（POD12）定义为接受一线治疗12个月内出现疾病进展，POD24定义为接受一线治疗24个月内出现疾病进展[18]。无进展生存（PFS）期定义为从疾病明确诊断开始至疾病进展或末次随访时间，总生存（OS）期定义为从疾病明确诊断开始到任何原因死亡或末次随访时间[19]。

4. 统计学处理：计数资料用例数（百分比）表示，计量资料用*M*（范围）表示，分类变量使用*χ*^2^检验或Fisher确切概率法进行检验，使用Mann-Whitney *U*或独立样本*t*检验对连续变量进行分析。使用Kaplan-Meier法估计生存率，制作生存曲线，并通过对数秩检验评估差异。采用Logistic回归对IGHV3-48与临床特征进行相关性分析，OS和PFS使用Cox回归模型进行单因素和多因素分析。当*P*<0.05时，差异被认为具有统计学意义。所有分析均使用SPSS 27.0.1软件进行。

## 结果

1. 临床特征：本研究共纳入33例患者，中位年龄为56（33～73）岁，年龄≥60岁患者占36.4％，女性患者占45.5％，所有患者均有骨髓浸润、Ann Arbor分期为Ⅳ期。本研究中FLIPI-1高危患者占62.5％。共有30例（90.9％）患者接受了治疗，治疗方案中均包含抗CD20单克隆抗体。3例患者自诊断至末次随访未接受治疗。患者的临床特征见表1。

**表1:** 33例滤泡性淋巴瘤患者的临床特征［例（％）］

临床特征	统计值
年龄［岁，*M*（范围）］	56（33～73）
年龄≥60岁	12（36.4）
性别	
女性	15（45.5）
男性	18（54.5）
B症状	13（39.4）
ECOG评分≥2分	1（3.0）
骨髓浸润	33（100）
组织学分级^a^	
Ⅰ～Ⅱ级	27（84.4）
Ⅲ A级	5（15.6）
FLIPI-1评分^a^	
0～1分（低危）	1（3.1）
2分（中危）	11（34.4）
≥3分（高危）	20（62.5）
FLIPI-2评分^a^	
0分（低危）	0（0）
1～2分（中危）	18（56.3）
≥3分（高危）	14（43.8）
Ann Arbor分期（Ⅳ期）	33（100）
一线治疗方案	
未治疗	3（9.1）
G/R-蒽环类药物	18（54.6）
G/R-苯达莫司汀	8（24.2）
G/R±无化疗方案	4（12.1）

**注** B症状：持续发热、体重减轻、盗汗；ECOG：美国东部肿瘤协作组；FLIPI：滤泡性淋巴瘤国际预后指数；G：奥妥珠单抗；R：利妥昔单抗。^a^共有32例（97.0％）患者具有组织学分级、FLIPI-1或FLIPI-2评分数据

2. FL中IGHV突变状态和VH CDR3长度：本研究中33例患者均存在免疫球蛋白重链基因重排。研究结果显示，33例患者中同源性百分比范围为83.1％～99.3％，中位胚系基因符合率为90.7％。32例（97.0％）IGHV突变，仅1例患者（IGHV4亚组）IGHV无突变（同源符合率99.3％）。各IGHV亚组间的SHM比例差异无统计学意义（*P*值均>0.05），IGHV1（1/1，100％）、IGHV2（1/1，100％）、IGHV3（23/23，100％）及IGHV5（1/1，100％）病例均为SHM，仅IGHV4亚组中1例未检测到SHM（阳性率为85.7％，6/7）。免疫球蛋白重链可变区互补决定区3（VH CDR3）中位长度为14（5～21）个氨基酸，各亚组间的VH CDR3平均氨基酸长度差异均无统计学意义（*P*值均>0.05）。

3. FL中IGHV、IGHD和IGHJ亚组及基因使用情况：如表2所示，在33例患者中，我们共鉴定出15种功能性IGHV基因。其中，IGHV3家族在患者群体中呈现显著优势分布（23例，69.7％），使用频率高的基因片段为IGHV3-48（9例，27.3％）、IGHV3-23（4例，12.1％）和IGHV3-66（4例，12.1％）。在IGHD基因中，最常见的亚组为IGHD3（11例，34.4％）、IGHD2（8例，25.0％）和IGHD4（5例，15.6％），其中使用频率高的基因片段为IGHD2-21（3例，9.4％）、IGHD3-16（3例，9.4％）和IGHD3-22（3例，9.4％）。此外，IGHJ基因中最常见的是IGHJ4-2（22例，66.7％）。在对各基因片段间的重排协作关系进行分析时发现IGHV3家族与IGHD3、IGHJ4家族之间存在频繁的配对使用，其中IGHV3-IGHD3-IGHJ4为该队列中最常见的V-D-J重排组合模式（图1）。

**表2:** 33例滤泡性淋巴瘤患者免疫球蛋白重链可变区（IGHV）基因谱分析

IGHV基因	例（％）
V区片段	
1	1（3.0）
2	1（3.0）
3	23（69.7）
4	7（21.2）
5	1（3.0）
V区主要基因	
1-69	1（3.0）
2-5	1（3.0）
3-11	1（3.0）
3-15	1（3.0）
3-21	1（3.0）
3-23	4（12.1）
3-30	1（3.0）
3-48	9（27.3）
3-64D	1（3.0）
3-66	4（12.1）
3-74	1（3.0）
4-34	3（9.1）
4-39	2（6.1）
4-59	2（6.1）
5-51	1（3.0）
D区主要基因^a^	
2-21	3（9.4）
3-16	3（9.4）
3-22	3（9.4）
J区主要基因	
4-2	22（66.7）
5-2	3（9.1）
6-2	5（15.2）

**注** V：可变区；D：多样性；J：连接区；a：IGHD基因数据有效例数为32例

**图1:**
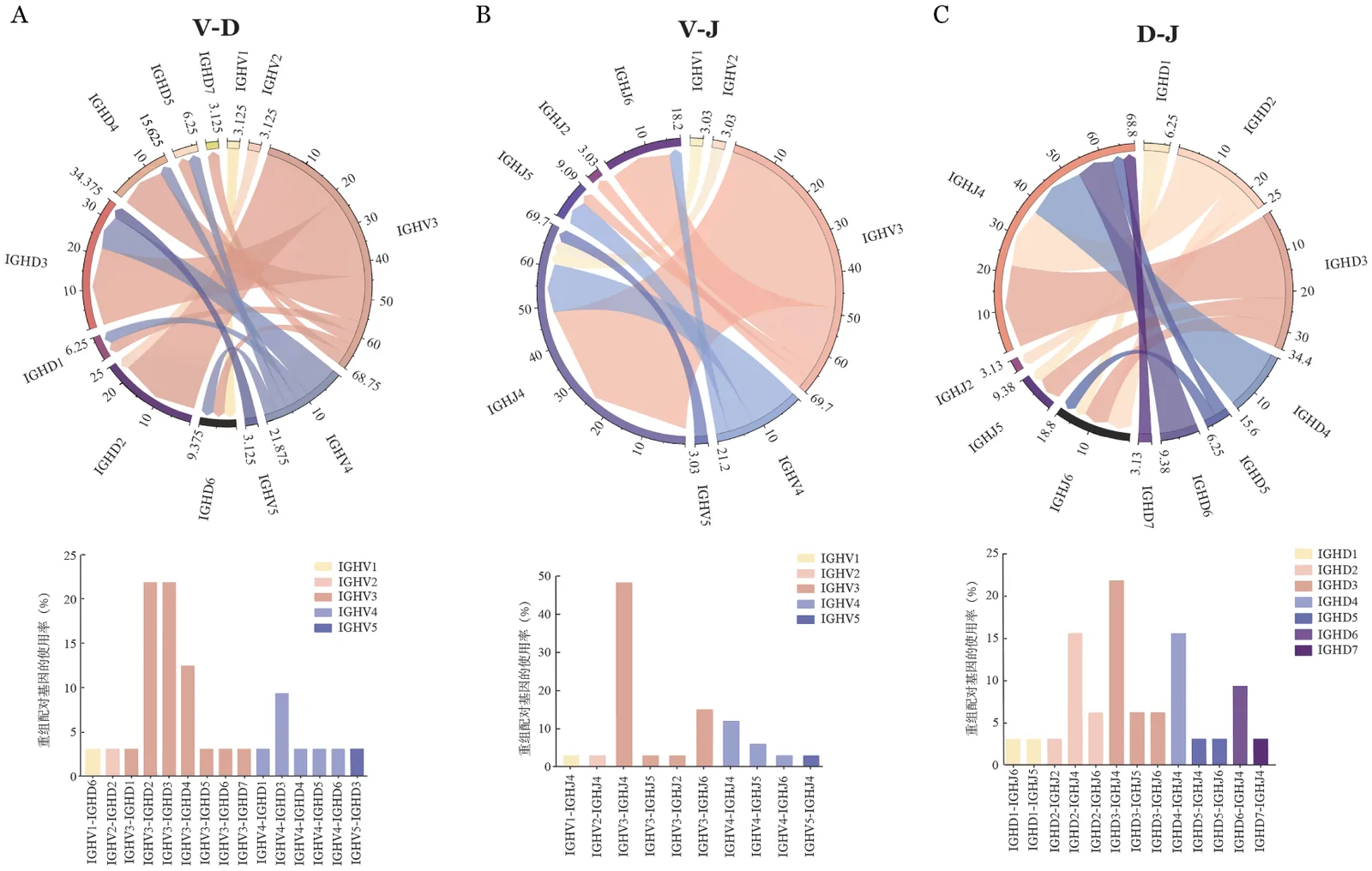
滤泡性淋巴瘤患者免疫球蛋白重链可变区（IGHV）-多样性（D）-连接区（J）基因在亚组层面的重排协作关系Circos图 **A** IGHV与IGHD基因在亚组层面的重排协作关系；**B** IGHV与IGHJ基因在亚组层面的重排协作关系；**C** IGHD与IGHJ基因在亚组层面的重排协作关系 **注** V-D：IGHV与IGHD重排协作；V-J：IGHV与IGHJ重排协作；D-J：IGHD与IGHJ重排协作；柱状图显示各IGHV或IGHD基因亚组（X轴）与其重组配对基因的使用百分比（Y轴）

4. FL与其他B细胞淋巴增殖性疾病（B-LPD）的IGHV使用特征的比较分析：整合国内多家中心报道的其他B-LPD的IGHV数据并与本研究的FL数据集进行分析。纳入比较的其他B-LPD包括CLL（595例）[20]、SMZL（40例）[21]、华氏巨球蛋白血症（WM）（44例）[22]和毛细胞白血病（HCL）（29例）[23]。结果显示IGHV3家族在FL中的使用频率（69.7％）高于除WM外的其他B-LPD（CLL：69.7％对51.2％，*P*＝0.038；SMZL：69.7％对20.5％，*P*<0.001；HCL：69.7％对31.0％，*P*＝0.002）。FL患者IGHV1家族的使用频率低于SMZL（3.0％对41.0％，*P*<0.001）和HCL（3.0％对20.7％，*P*＝0.044）。此外，FL患者IGHV3-48基因的使用频率高于其他B-LPD患者（CLL：27.3％对3.8％，*P*<0.001；SMZL：27.3％对0，*P*<0.001；WM：27.3％对2.2％，*P*<0.001；HCL：27.3％对6.9％，*P*＝0.048）。

5. IGHV家族和基因片段对临床结局的影响与生存分析：中位随访时间为41.5（8～133）个月。单因素分析中，IGHV各家族与OS均无明显相关性（*P*>0.05）。如表3所示，IGHV3-48基因显著富集于LDH升高［66.7％对13.6％；*OR*＝12.7（95％*CI*：1.6～102.3），*P*＝0.017］和FLIPI-2评分≥3分［88.9％对33.3％；*OR*＝15.0（95％ *CI*：1.6～142.2），*P*＝0.018］的患者中；此外，虽然差异无统计学意义，但IGHV3-48基因使用的患者POD12的风险高于其他IGHV基因使用的患者［44.4％对12.5％；*OR*＝4.8（95％ *CI*：0.8～28.9），*P*＝0.087］。受限于样本量，多因素分析中IGHV3-48与临床结局的相关性未得到证实。

**表3:** IGHV3-48与滤泡性淋巴瘤患者临床特征的单因素分析

变量	*OR*（95％ *CI*）	*P*值
年龄（≥60岁）	0.4（0.1～2.4）	0.310
性别	0.6（0.1～2.7）	0.478
PD	1.8（0.4～8.2）	0.478
POD12	4.8（0.8～28.9）	0.087
POD24	3.4（0.6～18.8）	0.160
B症状	2.5（0.5～12.0）	0.251
LDH	12.7（1.6～102.3）	0.017
β_2_微球蛋白	2.3（0.4～13.4）	0.372
FLIPI-1评分	7.3（0.8～68.5）	0.080
FLIPI-2评分	15.0（1.6～142.2）	0.018
染色体异常	0.5（0.1～3.0）	0.429
淋巴结直径≥6 cm	4.0（0.7～21.8）	0.109

**注** IGHV：免疫球蛋白重链可变区；PD：疾病进展；POD12：接受一线治疗12个月内出现疾病进展；POD24：接受一线治疗24个月内出现疾病进展；LDH：乳酸脱氢酶；B症状：持续发热、体重减轻、盗汗；ECOG：美国东部肿瘤协作组；FLIPI：滤泡性淋巴瘤国际预后指数

生存分析方面，单因素分析显示B症状［*HR*＝3.4（95％ *CI*：1.1～10.5），*P*＝0.035］和Ki-67指数≥30％［*HR*＝4.6（95％ *CI*：1.2～17.2），*P*＝0.022］与PFS相关。然而，多因素Cox回归分析中，上述所有因素（包括IGHV3-48、B症状及Ki-67指数≥30％）均未显示出独立的统计学意义。此外，所有因素与患者OS均无相关性（*P*值均>0.05）。

## 讨论

FL作为一种生物学和临床异质性疾病，目前尚无法治愈，临床特点是反复复发和缓解[24]。部分高危患者易早期进展或转化为侵袭性淋巴瘤，预后不佳[25]。本研究分析了伴有骨髓浸润的FL患者中IGHV基因的使用和突变状态及相关因素。首先，大部分患者采用覆盖IGHV区全长的leader引物成功扩增，97％患者为IGHV突变，与FL生发中心来源的本质一致[26]。其次，我们对IGHV、IGHJ、IGHD各家族进行分析，结果显示，IGHV3、IGHD3及IGHJ4为克隆性V-D-J重排序列中各主要亚型。在随后的临床相关性分析中，尽管未发现任何IGHV基因家族与患者临床结局之间存在显著关联，但观察到IGHV基因的使用存在偏移。IGHV3家族在FL中的使用率最高，其中IGHV3-48基因使用占比最高（27.3％），且IGHV3-48基因使用频率高于其他B-LPD（*P*值均<0.01）；而IGHV3-48基因使用与LDH水平升高和FLIPI-2评分≥3分存在相关性，且IGHV3-48基因使用的患者POD12的风险可能更高。

本研究FL患者IGHV1家族基因的使用频率仅为3.0％，显著低于IGHV3家族基因的使用频率（69.7％）。这一发现与Berget[9]和García-Álvarez等[10]的研究结果一致，但与Catherwood等[27]的报道存在差异。尽管IGHV3家族的整体使用频率与上述研究相似，但具体基因分布存在差异。本研究中，IGHV3-48基因的使用占比最高，而上述研究则分别以IGHV3-23和IGHV4-34基因为主。这种差异可能与本研究仅纳入伴有骨髓浸润的晚期FL患者有关。值得注意的是，Wartenberg等[25]报道的3例伴有骨髓浸润的FL患者均显示出IGHV3家族基因的使用，而Leich等[28]的研究则表明，IGHV4-34基因的使用在Ⅲ/Ⅳ期FL中更为常见。以上提示，FL患者的IGHV基因使用模式可能受到疾病分期、骨髓浸润状态以及地域等多种因素的影响。未来需扩大样本量并纳入更多临床变量，以更全面地揭示FL中IGHV基因使用的异质性及其临床意义。

FL细胞通过持续的SHM在其IGHV基因中引入N-糖基化位点。这些位点在正常B细胞中极为罕见，但在FL中常见。研究表明，N-糖基化位点在FL的早期克隆中便已获得，并在疾病进展过程中保持高度稳定；无论是在诊断、复发还是转化事件中，N-糖基化位点在大多数亚克隆中均被保留，表明这些位点对肿瘤细胞的生存和扩散至关重要，是FL细胞与微环境相互作用的关键机制[29]–[32]。进一步的研究揭示，t（14;18）阳性的FL倾向于使用IGHV3-23、IGHV3-48等基因，并在CDR3区域获得N-糖基化位点，肿瘤细胞通过N-糖基化的低亲和力免疫球蛋白与树突状细胞或巨噬细胞相互作用，从而逃避凋亡，维持生存[28]–[29]。相比之下，t（14;18）阴性的FL则更倾向于使用IGHV4-34基因，并在FR3区域获得N-糖基化位点。这些细胞通过与自身抗原或异源抗原的相互作用来刺激B细胞受体，进而维持其生存[28]。单细胞测序数据进一步表明，IGHV区域N-糖基化位点的获得可驱动FL细胞从生发中心明区（LZ）样表型向暗区（DZ）样表型转变。这种表型转变与FL的克隆进化密切相关，也提示N-糖基化位点在FL的疾病进展中扮演着关键角色[33]–[34]。

尽管本研究未提供IGHV基因N-糖基化位点的相关数据，但在复发模式的分析中观察到使用IGHV3-48基因的患者中有55.6％出现了疾病复发。此前的研究表明，IGHV3-48基因在发生组织学转化（HT）的FL病例中显著富集（19％），而在未发生HT的FL病例中，IGHV3-48的使用率仅为5％；此外，使用IGHV3-48基因的患者在10年内发生HT的概率显著高于使用其他基因的患者（71％对25％）[10]。由于随访时间有限、样本量较小等因素，本研究队列仅观察到1例患者（非IGHV3-48）出现HT的现象，但IGHV3-48基因的使用者作为早期进展的高危患者，仍需引起临床上的高度重视。

综上，本研究提示IGHV3-48基因在伴骨髓浸润的高危FL患者中显著富集，其对该类患者的早期预后预测可能具有一定参考价值，或可为后续临床预测指标的探索提供线索与方向。本研究纳入的样本量有限，统计检验效力不足。此外，本研究中与其他B-LPD队列的比较为描述性、探索性比较，其目的仅为初步揭示不同B细胞淋巴瘤亚型间免疫球蛋白基因重排的分布差异，不能等同于严格的直接对照研究。不同队列的入组标准、检测方法、时间跨度及临床特征均有显著差异。因此，目前所得结论主要为初步探索，其确切临床意义需进一步扩大研究规模进行验证。另外，本研究主要聚焦于具有骨髓浸润的高危FL患者，未纳入低危或无骨髓浸润的FL患者，因此无法全面评估IGHV3-48基因使用对FL患者整体预后的影响。未来需要通过扩大样本量，进一步明确IGHV3-48基因在FL中的生物学意义及其对患者预后的影响。
